# Neighborhood Influences on Violent Reoffending Risk in Released Prisoners Diagnosed With Psychotic Disorders

**DOI:** 10.1093/schbul/sbx071

**Published:** 2017-06-01

**Authors:** Amir Sariaslan, Henrik Larsson, Paul Lichtenstein, Seena Fazel

**Affiliations:** 1 Department of Psychiatry, University of Oxford, Warneford Hospital, Oxford, UK;; 2 Department of Medical Epidemiology and Biostatistics, Karolinska Institutet, Stockholm, Sweden;; 3 School of Medical Sciences, Örebro University, Örebro, Sweden

**Keywords:** causal inference, natural experiments, neighborhood effects, psychotic disorders, substance use disorders, socioeconomic status, violence

## Abstract

Released prisoners diagnosed with psychotic disorders have elevated rates of violent reoffending risk and their exposure to adverse neighborhood environments may contribute to this risk. We identified all released sentenced prisoners in Sweden between 2003 and 2013 (*n* = 47226) and followed them up for a median period of 4.4 years. We identified prisoners who had ever been diagnosed with a psychotic disorder (*n* = 3782) or prescribed antipsychotics (*n* = 7366). We examined 3 neighborhood characteristics: income, proportion of welfare recipients, and crime rate. By fitting generalized mixed-effects and negative binomial regression models and adopting within-individual designs that controlled for all time-invariant unmeasured confounders within each individual, we estimated neighborhood intraclass correlations (ICCs) and associations between specific neighborhood characteristics and violent reoffending. Neighborhood factors explained 13.5% (95% CI: 10.9%; 16.6%) of the violent reoffending risk among released prisoners diagnosed with psychotic disorders. This contrasted with 4.3% (95% CI: 3.7%; 4.9%) in all released prisoners. However, after controlling for unmeasured confounding, these estimates were not statistically significant (ICC_psychotic disorders_ = 0.9%; 95% CI: −0.8%; 2.3%; ICC_all prisoners_ = 0.3%; 95% CI: −0.02%; 0.6%). Similarly, none of the within-individual correlations between the specific neighborhood factors and violent reoffending were significantly different from zero. We found consistent results when we investigated prisoners with other psychiatric and substance use disorders. These findings suggest that placing released prisoners with psychotic disorders in less deprived neighborhoods might not reduce their violent reoffending risk, which may also apply to other psychiatric disorders. The assessment, treatment, and community linkage of high-risk prisoners as a strategy to reduce reoffending needs further research.

## Introduction

The global prison population reached an estimated 10.4 million individuals in 2015, which represents a 20% increase since 2000.^1^ Nearly 30 million individuals enter and leave prisons each year across the world.^2^ Around two-thirds of released prisoners in the United States are rearrested within 3 years,^3,4^ with similarly high reoffending rates reported in other high income countries.^5^ High reoffending rates have been attributed to concentrating released prisoners in socioeconomically deprived neighborhoods, characterized by poor employment opportunities, weak public institutions (eg, schools and community organizations), and increased residential mobility and antisocial behaviors.^6^ Although some evidence suggests that neighborhood factors predict reoffending,^7–9^ it is uncertain whether neighborhood influences affect former prisoners with psychotic disorders who reoffend at higher rates than other prisoners.^10,11^ Expert opinion suggests that violence prevention efforts in released prisoners should not only focus on treating mental illnesses and substance use disorders but also address a wider range of socioeconomic factors.^12,13^ But their effectiveness will depend on whether associations between socioeconomic status and violent reoffending are consistent with a causal inference. To date, this has been lacking.

To address methodological limitations of previous research, we used nationwide registry data on a sample of all Swedish prisoners who were released between 2003 and 2013 (*n* = 47226). We were able to obtain annual measures of their residential areas throughout the entire follow-up period, which allowed us to examine the time-varying associations between 3 objective measures of neighborhood characteristics from high-quality registers, namely the median income, proportion of individuals receiving means-tested welfare benefits, and crime rate, and subsequent violent reoffending. In complementary analyses, we also considered 2 alternative measures of socioeconomic status: disposable income and welfare recipiency. To account for unmeasured confounders, we adopted, for the first time to our knowledge, a within-individual research design, where each individual served as their own controls across time, which allowed us to indirectly control for an aggregate of all of their time-constant confounders (eg, genetic and early childhood risks). The large and representative sample further allowed us to examine subsamples of individuals who had ever been diagnosed with or prescribed medication for psychotic disorders. In addition, the sample allowed us to compare the latter findings with a large number of other psychiatric and substance use disorders.

## Methods

### Study Setting

We linked 8 nationwide high-quality registers. The Prison and Probation Services Register provided dates of imprisonment in Swedish prisons between 2003 and 2013. We obtained dates of violent crime convictions from the National Crime Register, which includes all criminal convictions in lower general court in Sweden since 1973. Data on residential areas were gathered from the Small Area Marketing Statistics (SAMS) Register, which is maintained by Statistics Sweden and includes geo-coded data for all individuals. The geo-coded data are based on the registered addresses of all Swedish residents retrieved from the National Tax Agency at the end of each year. The coverage is nearly complete as it is legally mandatory for all residents to register their physical address with the agency. The Integrated Database for Labor Market Research provided annual data on sociodemographic factors between 1990 and 2013 for all individuals aged more than 16, who were alive and registered as residents at the end of each year. Emigration and mortality dates came from the Migration and the Causes of Death Registers, respectively. The Prescribed Drug Register included data on all dispensed prescription drugs with Anatomical Therapeutic Chemical (ATC) codes between July 1, 2005 and December 31, 2013. The National Patient Register (NPR) provided data on all inpatient hospitalization episodes (ICD-8, -9, and -10; 1973–2013) and specialist outpatient care visits (ICD-10; 2001–2013). The NPR is comprehensive to the universal health coverage in Sweden.

All Swedish residents are assigned a unique 10-digit civic registration number, either at birth or upon immigration, which is used in all nationwide registers, and accurate linkage. We selected a cohort of all released prisoners who had served any number of prison sentences between January 1, 2003 and December 31, 2013. All individuals were followed up from their date of prison release up until migration, re-incarceration, death or end of follow-up (December 31, 2013). If they had served multiple sentences, we included all of their person-time in the community and censored them according to the same criteria. Anonymized data was received from Statistics Sweden following Regional Research Ethics Committee approval at Karolinska Institutet (2013/5:8).

## Measures

### Neighborhood Definition

In keeping with previous population-based research,^14^ we used the SAMS definition of Swedish neighborhoods, which identifies approximately 9200 geographically small and socioeconomically homogenous residential areas based on the local housing profile. The average population size of SAMS neighborhoods is about 1000 individuals, which corresponds to a quarter of that of the average census tract in the United States.^15^ Smaller neighborhood definitions tend to increase the explained variation in criminal offending by maximizing the within-neighborhood similarities between the residents.^16^ Qualitative validation studies have demonstrated that the SAMS neighborhood definition strongly overlaps with public perceptions of neighborhood boundaries.^17^ There are considerable socioeconomic status differences across Swedish neighborhoods; the prevalence rate of welfare recipiency is 30 times more common in most deprived compared with most affluent neighborhoods.^18^

### Exposure Variables

We examined 3 annual measures of neighborhood characteristics that we generated by aggregating data on all residents aged 25–64 years. Neighborhood income was defined as the standardized, and reverse-coded, median disposable income (eg, net sum of all earnings and benefits). Neighborhood welfare measured the standardized prevalence rate of residents who had received means-tested welfare benefits at least once during the year. Neighborhood crime was defined as the number of criminal convictions in a given year divided by the number of neighborhood residents. All 3 measures were treated as continuous variables in the statistical analyses with the exception of the descriptive tables, where we present them as tertiles.

We additionally examined 3 alternative exposure variables that were not measured on the neighborhood level—disposable income, welfare benefits, and residential relocations. Disposable income was defined as the standardized, and reverse-coded, annual disposable income. Welfare benefits referred to the individual being granted means-tested welfare benefits during a given year. Residential relocations measured the number of home address changes reported to the National Tax Agency during a given year.

### Violent Reoffending

Consistent with previous studies,^19^ we defined violent criminality as homicide, assault, robbery, threats and violence against an officer, gross violation of a person’s integrity, unlawful threats, unlawful coercion, kidnapping, illegal confinement, arson, intimidation, or sexual offenses (rape, indecent assault, indecent exposure, or child molestation, but excluding prostitution, hiring of prostitutes, or possession of child pornography).

### Psychotic, Other Psychiatric, and Substance Use Disorders

Individuals who had ever been diagnosed with a schizophrenia-spectrum disorder (ICD-8: 295, 297–299; ICD-9: 295, 297–298; ICD-10: F20–F29) or bipolar disorder (ICD-8: 296 [excl. 296.2]; ICD-9: 296 [excl. 296B]; ICD-10: F30–F31) as an inpatient (1973–2013) or outpatient (2001–2013) were defined as having any psychotic disorder. We further included individuals that had ever been diagnosed with any personality disorder (ICD-8 and ICD-9: 301; ICD-10: F60–F69), unipolar depression (ICD-8: 296.2, 300.4; ICD-9: 296B, 300E, 311; ICD-10: F32–F39 [excl. F32.3]), anxiety (ICD-8: 300 [excl. 300.4]; ICD-9. 300 [excl. 300E]; ICD-10: F40–F48 [excl. F43]), alcohol use disorder (ICD-8: 291, 303; ICD-9: 291, 303, 305A; ICD-10: F10) and drug use disorder (ICD-8: 292, 304; ICD-9: 292, 304, 305X; ICD-10: F11–F12, F14–F16, F19). The Swedish registries have been extensively used to study a wide range of psychiatric disorders and external validation studies have confirmed that even single episode diagnoses of schizophrenia,^20^ bipolar disorder,^21^ personality disorder,^22^ depression,^23^ anxiety,^24^ and substance use disorders^25^ are valid. In complementary analyses, we also included individuals who had ever been prescribed antipsychotics (ATC: N05A [excl. N05AN01]), mood stabilizers (ATC: N03AF01-N03AF02, N03AG01, N03AX09, N05AN01), anxiolytics (ATC: N05B), antidepressants (ATC: N06A) and drugs used in alcohol dependence (ATC: N07BB), and opioid dependence (ATC: N07BC) between 2005 and 2013.

### Analytic Approach

We estimated neighborhood intraclass correlations (ICCs) for violent reoffending as an ordered categorical measure by fitting a series of generalized linear mixed-effects models (GLMMs).^26^ The neighborhood ICC statistic ranges between 0 and 1 and expresses the proportion of variance in violent reoffending risk that is attributed to neighborhood influences.^27^ Standard approaches to estimating neighborhood ICCs are typically biased because they make the implicit assumption that the choice of neighborhood residence is randomly distributed in the population. Our complex data structure on individuals who moved between different neighborhoods across time, allowed us to address this limitation by controlling for nonrandom self-selection patterns. To estimate the magnitude of this bias, we fitted 2 separate models that estimated the crude and the within-individual neighborhood ICCs. We fitted the crude models in Stata 14 MP (meologit) and the within-individual models in Mplus 7.4 (TYPE=CROSSCLASSIFIED).

To quantify the associations between each neighborhood exposure variable and violent reoffending, we estimated incidence rate ratios (IRRs) and corresponding 95% CIs by fitting 2 separate negative binomial regression models.^28^ The negative binomial distribution is preferred over the Poisson distribution when count outcome variables are over-dispersed due to a preponderance of zeros, which violates an assumption of the latter by inflating the variance relative to the mean. We allowed for the exposure variables to vary annually across the follow-up period and applied cluster-robust standard errors to account for the nested data structure of measurement occasions being nested within individuals. In Model I, we estimated the crude effect of each exposure variable on violent reoffending.

To examine the causal nature of the crude associations, we subsequently examined the within-individual associations between exposure variables and reoffending risk. The rationale for this natural experimental approach is to make the individuals serve as their own controls across time, thus controlling for an aggregate of all time-constant confounders in each individual (eg, all genetic and early environmental influences). This means that measured time-constant confounders, such as sex and immigrant background, are controlled for and do not need to be included in the models. Consistent with the between-within decomposition approach,^29^ we partitioned the exposure variables into a time-constant mean effect, known as the “between-individual effect,” and the time-varying deviations from the mean, known as the “within-individual effect.” For the associations to be consistent with a causal inference, one would assume that an individual who is, relative to their own mean (eg, the between-effect), exposed to higher levels of deprivation at specific time points (eg, the within-effect) to be more likely to violently reoffend. The Model II estimates refer to the within-individual, and age-adjusted, estimates. We fitted Models I–II in Stata 14 MP using the nbreg command.

### Sensitivity Analyses

To test for potential confounding by gender, ethnicity, and population size, we re-ran Models I–II on subsamples excluding females, individuals with an immigrant background, and those who lived in neighborhoods with less than 1500 inhabitants. We also tested whether individuals who moved between different neighborhoods across time were generalizable to the entire population of prisoners by comparing the age-adjusted rates of violent reoffending between the groups. We subsequently examined the effects of the neighborhood exposures (eg, neighborhood income, welfare recipients, and crime rate) in residentially mobile individuals by re-running the models using the average values of the exposures for each neighborhood across the entire study period. By removing the annual fluctuations, only individuals who had moved between different neighborhoods were able to contribute to the latter models. We explored the extent to which the duration of exposure to the neighborhood characteristics predicted violent reoffending risks by modifying Model II to include an interaction term between each of the 3 neighborhood exposure variables and the duration of time (in days) that the individual had resided in the neighborhood. We further assessed the sensitivity of our neighborhood definition by considering larger geographical representations (eg, parishes, municipalities, and counties). We tested the generalizability of our findings by fitting GLMMs to estimate within-individual correlations for each exposure variable across the follow-up period, thus examining the extent to which individuals were exposed to varying socioeconomic levels. We used Bonferroni correction to minimize the risks of obtaining false-positive results.

## Results

We identified a total of 47226 individuals released from Swedish prisons during the study period and followed them up for a median of 4.4 years. The lifetime prevalence rate of any psychotic disorders was 8% (*n* = 3782), while the equivalent estimate for antipsychotic prescriptions exceeded 15% (*n* = 7366). About a third (34.4%) had a lifetime diagnosis of any psychiatric disorder as inpatients or outpatients (*n* = 16262), nearly half (49.2%) were diagnosed with substance use disorders by psychiatric services (*n* = 23212) and a majority (54.7%) had been prescribed psychiatric medications (*n* = 25831). The absolute rates of violent reoffending were elevated in psychiatric disorders, particularly in those diagnosed with psychotic and personality disorders, as well as in certain sociodemographic categories, including males, younger age groups, and during periods of disadvantage across all of the examined exposure variables (table 1). We observed a similar pattern of sociodemographic effects, albeit generally with higher absolute risks, in the subsamples of individuals diagnosed with psychotic, other psychiatric and substance use disorders (ST1–ST12).

**Table 1.** Sociodemographic Data on the Full Sample of Released Prisoners

	Number of Individuals	Number of Person-Years at Risk	Number of Violent Crimes	Violent Reoffending Rate per Person-Year [95% CI]
Total	47226	207501	29999	0.45 [0.45; 0.45]
Sex				
Male	43562	190125	29172	0.48 [0.48; 0.48]
Female	3664	17376	827	0.10 [0.10; 0.10]
Age groups				
15–19 years	1165	1014	540	2.11 [2.05; 2.18]
20–24 years	9601	19293	5906	0.98 [0.97; 0.98]
25–29 years	12487	30710	5552	0.58 [0.57; 0.58]
30–34 years	11249	27455	4365	0.50 [0.49; 0.51]
35–39 years	10175	24712	3729	0.48 [0.47; 0.49]
40–44 years	10549	26618	3362	0.34 [0.33; 0.34]
45–49 years	10447	26853	3061	0.31 [0.30; 0.31]
50–54 years	8117	20781	1919	0.26 [0.25; 0.26]
55–59 years	5533	14087	971	0.17 [0.16; 0.18]
60–64 years	3487	8878	401	0.09 [0.09; 0.10]
65+ years	2233	7101	193	0.04 [0.04; 0.05]
Neighborhood income				
Tertile 1 (high)	26683	69040	9350	0.40 [0.40; 0.41]
Tertile 2	29465	70368	10340	0.46 [0.46; 0.47]
Tertile 3 (low)	26138	68093	10309	0.48 [0.48; 0.49]
Neighborhood welfare				
Tertile 1 (low)	26424	70318	8560	0.37 [0.36; 0.37]
Tertile 2	28666	68818	10801	0.49 [0.48; 0.49]
Tertile 3 (high)	24550	68364	10638	0.49 [0.49; 0.50]
Neighborhood crime				
Tertile 1 (low)	29900	70184	9163	0.39 [0.39; 0.40]
Tertile 2	34356	69393	10051	0.46 [0.45; 0.46]
Tertile 3 (high)	30266	67923	10783	0.50 [0.49; 0.50]
Psychiatric disorders				
Psychotic disorder	3782	15756	4720	0.94 [0.93; 0.95]
Personality disorder	5255	22573	6987	0.94 [0.93; 0.95]
Anxiety	9173	39457	8837	0.69 [0.68; 0.69]
Depression	8087	35476	6426	0.58 [0.57; 0.59]
Alcohol use disorder	15103	66543	14566	0.67 [0.66; 0.67]
Drug use disorder	16164	72414	17272	0.70 [0.69; 0.70]
Individuals prescribed psychiatric medications				
Antipsychotics	7366	31458	8295	0.82 [0.81; 0.83]
Mood stabilizers	3796	16183	4057	0.72 [0.71; 0.73]
Anxiolytics	17137	76513	13398	0.55 [0.54; 0.55]
Antidepressants	17836	79032	13998	0.55 [0.55; 0.56]
Drugs used in alcohol dependence	6416	27748	6402	0.71 [0.70; 0.72]
Drugs used in opioid dependence	2172	11107	1862	0.47 [0.46; 0.48]

*Note*: Violent reoffending rates were estimated using negative binomial regression models to adjust for overdispersion.

We initially observed that neighborhood influences accounted for approximately 4% of the violent reoffending risk (ICC = 4.3%; 95% CI: 3.7%; 4.9%; table 2). After controlling for unmeasured confounding, this effect was not significantly different from zero (ICC = 0.3%; 95% CI: −0.02%; 0.6%; table 2). In psychotic disorders, the proportion of variation in violent reoffending risk explained by neighborhoods changed from 13.5% (95% CI: 10.9%; 16.6%) in the unadjusted model to a not statistically significant estimate of 0.9% (95% CI: −0.8%; 2.3%) in the within-individual model, and in those taking antipsychotics, the equivalent estimates were reduced from 6.9% (95% CI: 5.4%; 8.7%) to 0.4% (95% CI: −0.4%; 1.1%). In other words, by considering residential relocations in individuals who moved between different neighborhoods across time, we found no statistically significant contributions of neighborhood influences on violent reoffending risk.

**Table 2.** Violent Reoffending Neighborhood Intraclass Correlations (ICC) Stratified Across all Released Prisoners, Prisoners With Psychiatric and Substance Use Disorder, and Prisoners That Have Been Prescribed Medications for Psychiatric and Substance Use Disorders

	Crude	Within-Individual
	ICC [95% CI]	ICC [95% CI]
All prisoners	4.3% [3.7%; 4.9%]	0.3% [−0.02%; 0.6%]
Psychiatric disorders		
Any psychotic disorders	13.5% [10.9%; 16.6%]	0.9% [−0.8%; 2.3%]
Personality disorder	11.0% [9.1%; 13.3%]	0.4% [−0.8%; 1.6%]
Anxiety	7.3% [5.9%; 9.1%]	0.4% [−0.4%; 1.1%]
Depression	8.7% [6.9%; 11.0%]	0.2% [−0.5%; 0.8%]
Alcohol use disorder	7.6% [6.5%; 8.9%]	0.4% [−0.1%; 0.9%]
Drug use disorder	5.5% [4.6%; 6.5%]	0.4% [−0.1%; 1.0%]
Individuals prescribed psychiatric medications		
Antipsychotics	6.9% [5.4%; 8.7%]	0.4% [−0.4%; 1.1%]
Mood stabilizers	10.3% [8.0%; 13.2%]	0.6% [−1.0%; 2.0%]
Anxiolytics	6.7% [5.7%; 8.0%]	0.4% [−0.2%; 1.0%]
Antidepressants	6.0% [5.0%; 7.2%]	0.4% [−0.1%; 0.9%]
Drugs used in alcohol dependence	6.8% [5.1%; 9.0%]	0.1% [−0.4%; 0.7%]
Drugs used in opioid dependence	10.3% [7.0%; 15.0%]	0.3% [−1.6%; 2.0%]

The adjusted estimates in prisoners with other psychiatric and substance use disorders were similar in magnitude (table 2). There was no evidence that these findings were attributed to the choice of our geographical representation as larger areas (eg, parishes, municipalities, and counties) explained substantially smaller proportions of the risk differences in violent reoffending (ST13). Furthermore, we found no statistically significant (*P* = .136) differences in age-adjusted violent reoffending rates between those who relocated to different neighborhoods (*n* = 25696) compared to those who stayed in the same neighborhood (*n* = 21530) during follow-up.

**Fig. 1.**
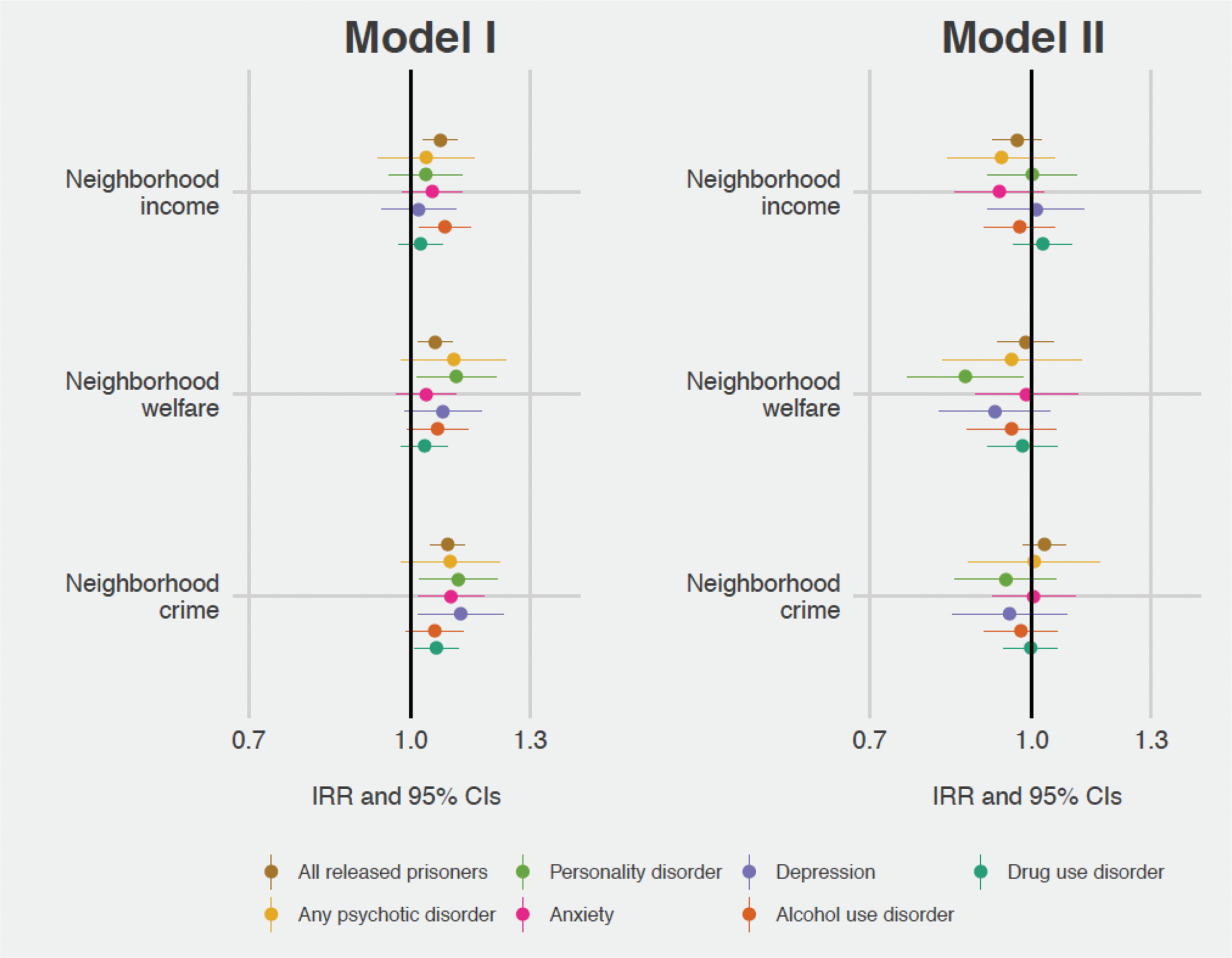
Incidence rate ratios for the associations between neighborhood factors stratified across prisoners that have been prescribed medications for psychiatric and substance use disorders. *Note*: Neighborhood income was reverse-coded (eg, the estimates refer to the effects on violent reoffending risk following a standardized unit reduction of the neighborhood income). Model I: crude between-estimate; Model II: within-individual estimate, adjusted for age. The Model II estimate for neighborhood welfare in personality disorders was not statistically significant following Bonferroni correction for multiple testing.

All of the specific neighborhood exposure variables predicted subsequent violent reoffending on the population level (figure 1). A standardized unit decrease in the median neighborhood income was, for instance, associated with a 7% increased reoffending rate (IRR = 1.07; 95% CI: 1.03; 1.11). This association was, however, entirely attenuated in the age-adjusted, within-individual model (IRR = 0.97; 95% CI: 0.92; 1.02). We found similar results across the remaining neighborhood factors and in the subsamples of individuals that met criteria for psychiatric and substance use disorders (figure 1 and table 3). We note that the inverse within-individual association between neighborhood welfare and violent reoffending in personality disorders (Model II, figure 1) was not statistically significant from zero following Bonferroni correction for multiple testing. Complementary sensitivity analyses using alternative exposure variables (eg, disposable income, welfare benefits, and residential relocations; table 4) and subsamples excluding females, individuals with immigrant backgrounds and neighborhoods with fewer than 1500 residents were consistent with the presented findings (ST14). Furthermore, we found no evidence that the associations between the neighborhood exposure variables and violent reoffending were moderated by the duration of stay in each neighborhood, as all of the interaction terms failed to reach statistical significance (all *P*-values exceeded .4). The within-individual correlations of exposure variables ranged between 0.28 and 0.68 (ST15), suggesting that the findings could not be attributed to insufficient variability in exposures across time. Lastly, we found that re-running the models using average values of the neighborhood exposures across the study period did not materially change the findings (ST16).

**Table 3.** Incidence Rate Ratios (IRRs) for the Associations Between Neighborhood Factors Stratified Across Prisoners That Have Been Prescribed Medications for Psychiatric and Substance Use Disorders

	Neighborhood Income		Neighborhood Welfare		Neighborhood Crime	
	Model I	Model II	Model I	Model II	Model I	Model II
	IRR [95% CI]	IRR [95% CI]	IRR [95% CI]	IRR [95% CI]	IRR [95% CI]	IRR [95% CI]
Antipsychotics	1.04 [0.97; 1.11]	0.97 [0.88; 1.06]	1.01 [0.95; 1.08]	1.00 [0.89; 1.12]	1.01 [0.95; 1.08]	1.07 [0.98; 1.17]
Mood stabilizers	1.04 [0.92; 1.17]	1.09 [0.95; 1.25]	1.02 [0.92; 1.13]	0.90 [0.77; 1.05]	1.09 [0.98; 1.20]	0.93 [0.83; 1.05]
Anxiolytics	1.05 [0.99; 1.11]	0.98 [0.91; 1.06]	1.04 [0.98; 1.10]	0.97 [0.89; 1.07]	1.03 [0.98; 1.09]	1.06 [0.99; 1.14]
Antidepressants	1.09 [1.03; 1.15]	0.93 [0.86; 1.00]	1.01 [0.96; 1.07]	0.96 [0.88; 1.06]	1.03 [0.98; 1.09]	1.05 [0.98; 1.12]
Drugs used in alcohol dependence	1.09 [1.00; 1.18]	0.93 [0.83; 1.04]	1.11 [1.00; 1.24]	0.96 [0.83; 1.12]	1.07 [0.98; 1.18]	1.05 [0.93; 1.19]
Drugs used in opioid dependence	0.95 [0.86; 1.05]	1.02 [0.90; 1.17]	1.05 [0.94; 1.16]	1.02 [0.87; 1.18]	1.07 [0.94; 1.22]	0.97 [0.84; 1.13]

*Note*: Neighborhood income was reverse-coded (eg, the estimates refer to the effects on violent reoffending risk following a standardized unit reduction of the neighborhood income). Model I: Crude between-estimate; Model II: Within-individual estimate, adjusted for age.

**Table 4.** Incidence Rate Ratios (IRRs) for the Associations Between Alternative Socioeconomic Status Exposure Variables (Disposable Income and Welfare Benefits), Residential Relocations and Violent Reoffending Stratified Across all Released Prisoners, Prisoners With Psychiatric and Substance Use Disorder, and Prisoners That Have Been Prescribed Medications for Psychiatric and Substance Use Disorders

	Disposable Income		Welfare Benefits		Residential Relocations	
	Model I	Model II	Model I	Model II	Model I	Model II
	IRR [95% CI]	IRR [95% CI]	IRR [95% CI]	IRR [95% CI]	IRR [95% CI]	IRR [95% CI]
All prisoners	1.36 [1.27; 1.45]	0.99 [0.92; 1.06]	2.81 [2.59; 3.04]	0.95 [0.86; 1.06]	1.30 [1.24; 1.36]	0.97 [0.92; 1.02]
Psychiatric disorders						
Any psychotic disorders	1.24 [1.07; 1.44]	0.92 [0.70; 1.20]	2.08 [1.67; 2.59]	0.98 [0.77; 1.24]	1.05 [0.95; 1.17]	1.07 [0.94; 1.20]
Personality disorder	1.27 [1.10; 1.48]	0.89 [0.74; 1.06]	1.95 [1.63; 2.32]	0.95 [0.77; 1.18]	1.16 [1.06; 1.26]	0.94 [0.86; 1.03]
Anxiety	1.18 [1.03; 1.34]	1.03 [0.91; 1.16]	1.81 [1.55; 2.12]	0.98 [0.82; 1.17]	1.19 [1.10; 1.28]	0.97 [0.88; 1.06]
Depression	1.27 [1.11; 1.46]	1.01 [0.83; 1.23]	2.05 [1.70; 2.46]	1.08 [0.87; 1.34]	1.23 [1.12; 1.34]	0.96 [0.86; 1.08]
Alcohol use disorder	1.20 [1.09; 1.33]	1.02 [0.91; 1.14]	2.29 [2.03; 2.58]	0.94 [0.80; 1.10]	1.27 [1.19; 1.35]	0.96 [0.89; 1.04]
Drug use disorder	1.07 [1.00; 1.15]	1.01 [0.93; 1.10]	1.76 [1.57; 1.97]	0.92 [0.80; 1.06]	1.17 [1.11; 1.24]	0.94 [0.88; 1.00]
Individuals prescribed psychiatric medications						
Antipsychotics	1.32 [1.18; 1.49]	0.97 [0.81; 1.16]	2.02 [1.70; 2.41]	0.95 [0.78; 1.16]	1.20 [1.11; 1.30]	0.96 [0.88; 1.06]
Mood stabilizers	1.14 [0.95; 1.38]	1.00 [0.87; 1.14]	1.97 [1.59; 2.46]	0.84 [0.67; 1.06]	1.17 [1.04; 1.32]	0.96 [0.83; 1.10]
Anxiolytics	1.28 [1.15; 1.43]	1.01 [0.92; 1.12]	2.38 [2.10; 2.70]	0.99 [0.85; 1.15]	1.27 [1.19; 1.36]	0.95 [0.88; 1.02]
Antidepressants	1.28 [1.16; 1.42]	1.01 [0.91; 1.11]	2.48 [2.19; 2.81]	0.91 [0.79; 1.05]	1.23 [1.15; 1.31]	0.99 [0.92; 1.07]
Drugs used in alcohol dependence	1.29 [1.13; 1.48]	1.07 [0.88; 1.30]	2.21 [1.83; 2.66]	1.08 [0.86; 1.35]	1.24 [1.13; 1.36]	1.02 [0.92; 1.13]
Drugs used in opioid dependence	1.23 [1.02; 1.49]	1.00 [0.77; 1.29]	1.45 [1.02; 2.06]	1.09 [0.69; 1.73]	1.24 [1.08; 1.42]	0.87 [0.73; 1.02]

*Note*: Disposable income was reverse-coded (eg, the estimates refer to the effects on violent reoffending risk following a standardized unit reduction of the disposable income). Model I: Crude between-estimate; Model II: Within-individual estimate, adjusted for age.

## Discussion

In this population-based longitudinal study of all 47226 released prisoners in Sweden between 2003 and 2013, we examined the extent to which neighborhood influences accounted for violent reoffending over a median follow-up of 4.4 years in prisoners with psychotic and other psychiatric disorders, and all prisoners. To our knowledge, this is the first study examining these associations by adopting a within-individual research design that accounts for time-constant unobserved confounders (eg, their genetic background and childhood environmental influences). The latter approaches allowed us to specifically examine the neighborhood effects on violent reoffending risk observed when individuals moved between different areas, and with high-quality neighborhood variables on 9200 different units, this provided stable estimates.

Our study has 3 principal findings. First, we initially observed that neighborhood influences explained a substantially larger proportion of the violent reoffending risks in released prisoners diagnosed with or prescribed medications for psychotic disorders than in the full sample of all released prisoners (7%–14% vs 4%). However, using within-individual designs, we found that the neighborhood correlations across all subgroups were not statistically significant from zero.

Second, this pattern was not different for other psychiatric disorders where high reoffending risk was initially observed in unadjusted models that attenuated to nil in within-individual designs.

Third, we observed that all three neighborhood exposure variables (median income, proportion of welfare recipients, and crime rate) independently predicted subsequent violent reoffending rates on the population level. For these associations to be consistent with a causal inference, we would have expected released prisoners to have higher rates of violent reoffending during periods when they resided in socioeconomically disadvantaged neighborhoods. However, once we had controlled for time-constant unobserved confounders, by re-examining the associations within individuals across time, there was no evidence of any statistically significant neighborhood effects. In other words, we found that the violent reoffending rates were constant regardless of where the released prisoners lived throughout the study period, so the neighborhood effects (if any) were likely minimal at best. Sensitivity analyses demonstrated that these findings were robust when using alternative exposure variables (disposable income and welfare recipiency) and could not be attributed to either confounding by gender, ethnicity, and neighborhood population size, duration effects, or the lack of variation in the exposure variables across time.

The latter findings, suggesting that the observed associations between socioeconomic status and violent reoffending are confounded, are generally consistent with randomized studies on housing and employment interventions for former prisoners^30,31^ as well as with 3 studies of adolescent offending.^18,32,33^ However, the results are not in keeping with influential studies of neighborhood effects on reoffending that have used inadequate controls for unmeasured confounders.^7–9^ This is important because studies examining employment effects on reoffending in former prisoners have shown that a sizable proportion of the variation in employment could potentially be attributed to selection factors.^34,35^ This is further supported by recent quantitative and molecular genetic studies that have demonstrated that adulthood residence in deprived neighborhoods is a considerably heritable trait that partly shares its genetic architecture with cognitive abilities, severe mental illnesses, and antisocial behaviors.^36,37^ Taken together, the current evidence underlines the importance of adequately addressing unmeasured confounding in studies of adverse outcomes in released prisoners and possibly other high-risk populations. Given the relative lack of such controls in the contemporary criminological literature examining the consequences of adverse neighborhood conditions,^38,39^ it could be the case, as some critics have argued,^18,40,41^ that such studies may have overemphasized the etiological role played by socioeconomic factors in explaining why violent criminality and other antisocial behaviors tend to aggregate within specific types of geographical areas.

If these findings are replicated in other contexts using similar designs and representative samples, it would suggest that efforts to support recently mentally ill released prisoners in securing housing in less deprived residential areas may not result in reduced violent reoffending. It is possible, however, that such interventions may have beneficial effects on other adverse outcomes, including violent victimization^42^ and unintended head injuries.^43^ Future work may benefit from combining large-scale registry data with within-individual research designs to test the causal nature of such associations. Nevertheless, the best current evidence suggests that violence prevention efforts in these groups should be continued by improving the assessment and treatment of higher risk prisoners who suffer from psychotic, other psychiatric, and substance use disorders.^10,11,44^ The timing such services is seemingly also of importance as a recent within-individual study on trigger factors for violent criminality in psychotic disorders demonstrated that the risk of violence sharply increased immediately following an exposure to a stressful life event (eg, violent victimization, parental bereavement, or an accident) as compared to earlier periods when the individuals were unexposed to the examined stressful life event.^45^

There are 6 main limitations to this study. First, the design relied on criminal conviction data to assess violent reoffending. Although this approach offers a comprehensive measure of severe violent acts, it will underestimate less severe cases. Therefore, it remains an empirical question whether our findings would be different if any criminal reoffending or violence that did not lead to convictions were used as outcomes. However, alternative methods commonly used to capture less severe offences, such as self-report, have other limitations including high rates of attrition and inflated measurement error, the latter of which contributes to artificial reductions of the true associations, especially in within-individual designs.^46^ Conviction data, on the other hand, partly reflects criminal justice practices, which is liable to detection bias in the most socioeconomically disadvantaged neighborhoods. This means, however, that our estimates are likely biased upwards. Second, recent estimates indicate that fewer than 0.5% of the Swedish population suffer from acute homelessness^47^ and are therefore not captured by our neighborhood definition. To examine the extent to which this biased our findings, we sensitivity tested and were able to fully replicate our main findings by using 2 alternative measures of socioeconomic status that were not neighborhood-specific, namely disposable income and welfare recipiency. Our findings cannot therefore be attributed to our inability to follow-up acutely homeless individuals.

Third, we were unable to measure residential moves to specific neighborhoods that had occurred within each year because the neighborhood data were derived from the National Tax Agency’s records at the end of each year. We were, however, able to measure the number of registered address changes within each year but we did not find that it predicted violent reoffending risk once unmeasured confounders were controlled for (table 4). Our findings could therefore not be attributed to higher residential mobility rates in former prisoners residing in deprived neighborhoods.

Fourth, we lacked sufficient statistical power to examine any potential differences between patients diagnosed with schizophrenia and bipolar disorder. Previous studies have demonstrated important etiological differences between these disorders with respect to their relationship with violent criminality.^48^ We note, however, that our findings were consistent across all of the examined categories of psychiatric disorders and prescription drugs. Similarly, we were underpowered to study any potential differences between specific types of violent crimes. Future replication efforts should therefore benefit from using larger samples with longer follow-up periods to examine whether the presented findings hold for individuals who commit different types of violent acts.

Fifth, services provided to prisoners once they are released vary within and between countries and we did not have access to such data in our sample. However, because our examined neighborhood factors did not predict violent reoffending rates within individuals across time, during periods where they had received different types of services, it suggests that such influences will likely be minimal on the examined associations.

Sixth, while there is considerable evidence that prisoner populations are similar across high-income countries,^11^ there are relatively smaller socioeconomic differences in Sweden, due to its comprehensive welfare state, than in some other high-income countries. It should be noted, however, that the socioeconomic differences in Sweden are still large with a 30-fold increased prevalence rate of welfare recipiency in the most deprived compared to the most affluent neighborhoods.^18^ Although comparative data are scarce, a previous criminological study concluded that the social mechanisms that increased the risk of violence across neighborhoods in Stockholm and Chicago were quite similar in nature, despite differences in the poverty rates (17.5% vs 6.3%).^49^ Similarly, we argue that larger socioeconomic status differences do not a priori imply causality; selection mechanisms should be explicitly modeled for before drawing such conclusions. We note that the crude neighborhood correlation of 4% reported here is similar in magnitude to similar studies, mostly conducted in the United States.^50^

In summary, we have demonstrated using a large representative sample of released prisoners that associations between neighborhood influences and later risks of violent reoffending were confounded by individual risks by using 2 different and complementary methodological approaches. Pending replication in other contexts, these findings suggest that efforts to assist mentally ill prisoners find housing in less deprived neighborhoods may not reduce violent reoffending. The role of assessing and treating higher risk prisoners with psychotic disorders in reducing serious reoffending needs urgent review.

## Supplementary Material

Supplementary data are available at *Schizophrenia Bulletin* online.

## Funding

The study was supported by the Wellcome Trust (095806), the Swedish Research Council for Health, Working Life and Welfare (project 2012-1678), and the Swedish Research Council (2011–2492).

## Supplementary Material

Supplementary_Tables
