# Supplementary material for: Neighborhood Influences on Violent Reoffending Risk in Released Prisoners Diagnosed With Psychotic Disorders
Source: Schizophr Bull. 2017 Jun 1;43(5):1011–20. doi: 10.1093/schbul/sbx071 (PMC5581887; doi:10.1093/schbul/sbx071)
Supplement: Supplementary_Tables [file sbx071_suppl_Supplementary_Tables.docx]

**ST1. Socio-demographic data on released prisoners with diagnoses of psychotic disorder.**

|  | **Number of individuals** | **Number of person-years at risk** | **Number of violent crimes** | **Violent reoffending**  **rate per person-year**  **[95% CI]** |
| --- | --- | --- | --- | --- |
| **Total** | 3782 | 15,756 | 4717 | 0.94 [0.93; 0.95] |
| **Sex** |  |  |  |  |
| Male | 3371 | 13,993 | 4513 | 1.02 [1.02; 1.02] |
| Female | 411 | 1763 | 207 | 0.27 [0.27; 0.27] |
| **Age groups** |  |  |  |  |
| 15-19 years | 68 | 51 | 52 | 6.45 [5.98; 6.95] |
| 20-24 years | 629 | 1201 | 694 | 1.74 [1.69; 1.80] |
| 25-29 years | 944 | 2161 | 756 | 1.19 [1.15; 1.23] |
| 30-34 years | 999 | 2341 | 815 | 1.23 [1.19; 1.26] |
| 35-39 years | 929 | 2127 | 702 | 0.99 [0.95; 1.03] |
| 40-44 years | 942 | 2235 | 538 | 0.60 [0.57; 0.63] |
| 45-49 years | 903 | 2233 | 544 | 0.62 [0.59; 0.65] |
| 50-54 years | 634 | 1518 | 329 | 0.74 [0.70; 0.78] |
| 55-59 years | 413 | 1000 | 193 | 0.54 [0.50; 0.59] |
| 60-64 years | 218 | 509 | 57 | 0.30 [0.25; 0.34] |
| 65+ years | 116 | 380 | 40 | 0.27 [0.22; 0.32] |
| **Neighborhood income** |  |  |  |  |
| Tertile 1 (high) | 2211 | 5226 | 1540 | 0.91 [0.89; 0.93] |
| Tertile 2 | 2369 | 5372 | 1647 | 1.00 [0.97; 1.02] |
| Tertile 3 (low) | 2181 | 5159 | 1533 | 0.91 [0.89; 0.94] |
| **Neighborhood welfare** |  |  |  |  |
| Tertile 1 (low) | 2148 | 5346 | 1370 | 0.78 [0.76; 0.80] |
| Tertile 2 | 2383 | 5215 | 1670 | 1.02 [0.99; 1.04] |
| Tertile 3 (high) | 2383 | 5196 | 1680 | 1.03 [1.00; 1.05] |
| **Neighborhood crime** |  |  |  |  |
| Tertile 1 (low) | 2413 | 5341 | 1454 | 0.83 [0.81; 0.85] |
| Tertile 2 | 2755 | 5262 | 1544 | 0.93 [0.91; 0.95] |
| Tertile 3 (high) | 2433 | 5153 | 1722 | 1.06 [1.04; 1.09] |

Note: Violent reoffending rates were estimated using negative binomial regression models to adjust for overdispersion.

**ST2. Socio-demographic data on released prisoners with diagnoses of personality disorder.**

|  | **Number of individuals** | **Number of person-years at risk** | **Number of violent crimes** | **Violent reoffending**  **rate per person-year**  **[95% CI]** |
| --- | --- | --- | --- | --- |
| **Total** | 5255 | 22,574 | 6987 | 0.94 [0.93; 0.95] |
| **Sex** |  |  |  |  |
| Male | 4618 | 19,699 | 6657 | 1.03 [1.03; 1.03] |
| Female | 637 | 2872 | 330 | 0.26 [0.26; 0.26] |
| **Age groups** |  |  |  |  |
| 15-19 years | 83 | 70 | 70 | 2.04 [2.04; 2.04] |
| 20-24 years | 915 | 1715 | 1174 | 1.68 [1.68; 1.68] |
| 25-29 years | 1391 | 3109 | 1229 | 1.38 [1.38; 1.38] |
| 30-34 years | 1435 | 3274 | 1152 | 1.13 [1.13; 1.13] |
| 35-39 years | 1395 | 3213 | 954 | 0.93 [0.93; 0.93] |
| 40-44 years | 1410 | 3375 | 815 | 0.76 [0.76; 0.76] |
| 45-49 years | 1312 | 3221 | 769 | 0.62 [0.62; 0.62] |
| 50-54 years | 933 | 2322 | 435 | 0.51 [0.51; 0.51] |
| 55-59 years | 538 | 1298 | 233 | 0.42 [0.42; 0.42] |
| 60-64 years | 265 | 620 | 134 | 0.34 [0.34; 0.34] |
| 65+ years | 121 | 356 | 22 | 0.28 [0.28; 0.28] |
| **Neighborhood income** |  |  |  |  |
| Tertile 1 (high) | 3130 | 7587 | 2201 | 0.87 [0.85; 0.89] |
| Tertile 2 | 3454 | 7700 | 2378 | 0.99 [0.97; 1.01] |
| Tertile 3 (low) | 3126 | 7286 | 2408 | 0.96 [0.94; 0.98] |
| **Neighborhood welfare** |  |  |  |  |
| Tertile 1 (low) | 3186 | 7628 | 2099 | 0.77 [0.75; 0.79] |
| Tertile 2 | 3362 | 7434 | 2474 | 1.02 [1.00; 1.04] |
| Tertile 3 (high) | 2868 | 7511 | 2414 | 1.03 [1.01; 1.05] |
| **Neighborhood crime** |  |  |  |  |
| Tertile 1 (low) | 3485 | 7629 | 2161 | 0.84 [0.82; 0.85] |
| Tertile 2 | 3917 | 7561 | 2281 | 0.89 [0.87; 0.91] |
| Tertile 3 (high) | 3466 | 7383 | 2545 | 1.09 [1.07; 1.11] |

Note: Violent reoffending rates were estimated using negative binomial regression models to adjust for overdispersion.

**ST3. Socio-demographic data on released prisoners with diagnoses of anxiety.**

|  | **Number of individuals** | **Number of person-years at risk** | **Number of violent crimes** | **Violent reoffending**  **rate per person-year**  **[95% CI]** |
| --- | --- | --- | --- | --- |
| **Total** | 9173 | 39,456 | 8837 | 0.69 [0.68; 0.69] |
| **Sex** |  |  |  |  |
| Male | 7986 | 34,038 | 8449 | 0.77 [0.77; 0.77] |
| Female | 1187 | 5419 | 388 | 0.15 [0.15; 0.15] |
| **Age groups** |  |  |  |  |
| 15-19 years | 192 | 163 | 113 | 1.64 [1.64; 1.64] |
| 20-24 years | 1913 | 3662 | 1725 | 1.29 [1.29; 1.29] |
| 25-29 years | 2693 | 6298 | 1668 | 1.02 [1.02; 1.02] |
| 30-34 years | 2508 | 5943 | 1542 | 0.80 [0.80; 0.80] |
| 35-39 years | 2248 | 5326 | 1103 | 0.63 [0.63; 0.63] |
| 40-44 years | 2239 | 5484 | 1001 | 0.50 [0.50; 0.50] |
| 45-49 years | 2084 | 5287 | 947 | 0.39 [0.39; 0.39] |
| 50-54 years | 1423 | 3658 | 418 | 0.31 [0.31; 0.31] |
| 55-59 years | 825 | 2000 | 206 | 0.25 [0.25; 0.25] |
| 60-64 years | 415 | 1002 | 74 | 0.19 [0.19; 0.19] |
| 65+ years | 209 | 634 | 40 | 0.15 [0.15; 0.15] |
| **Neighborhood income** |  |  |  |  |
| Tertile 1 (high) | 5367 | 13,163 | 2717 | 0.60 [0.59; 0.61] |
| Tertile 2 | 5920 | 13,380 | 3199 | 0.79 [0.78; 0.81] |
| Tertile 3 (low) | 5303 | 12,914 | 2921 | 0.67 [0.66; 0.68] |
| **Neighborhood welfare** |  |  |  |  |
| Tertile 1 (low) | 5444 | 13,323 | 2693 | 0.59 [0.58; 0.61] |
| Tertile 2 | 5756 | 13,126 | 3094 | 0.71 [0.70; 0.73] |
| Tertile 3 (high) | 5010 | 13,007 | 3050 | 0.75 [0.74; 0.76] |
| **Neighborhood crime** |  |  |  |  |
| Tertile 1 (low) | 6054 | 13,316 | 2749 | 0.59 [0.58; 0.60] |
| Tertile 2 | 6761 | 13,243 | 2951 | 0.67 [0.66; 0.68] |
| Tertile 3 (high) | 6003 | 12,897 | 3137 | 0.81 [0.79; 0.82] |

Note: Violent reoffending rates were estimated using negative binomial regression models to adjust for overdispersion.

**ST4. Socio-demographic data on released prisoners with diagnoses of depression.**

|  | **Number of individuals** | **Number of person-years at risk** | **Number of violent crimes** | **Violent reoffending**  **rate per person-year**  **[95% CI]** |
| --- | --- | --- | --- | --- |
| **Total** | 8087 | 35,476 | 6426 | 0.58 [0.57; 0.59] |
| **Sex** |  |  |  |  |
| Male | 7090 | 30,993 | 6159 | 0.65 [0.65; 0.65] |
| Female | 997 | 4544 | 267 | 0.11 [0.11; 0.11] |
| **Age groups** |  |  |  |  |
| 15-19 years | 125 | 118 | 71 | 1.36 [1.36; 1.36] |
| 20-24 years | 1320 | 2597 | 978 | 1.11 [1.11; 1.11] |
| 25-29 years | 1970 | 4716 | 1061 | 0.90 [0.90; 0.90] |
| 30-34 years | 1956 | 4790 | 1023 | 0.73 [0.73; 0.73] |
| 35-39 years | 1891 | 4420 | 958 | 0.59 [0.59; 0.59] |
| 40-44 years | 2001 | 4979 | 752 | 0.48 [0.48; 0.48] |
| 45-49 years | 2008 | 5118 | 738 | 0.39 [0.39; 0.39] |
| 50-54 years | 1528 | 3895 | 443 | 0.32 [0.32; 0.32] |
| 55-59 years | 978 | 2443 | 238 | 0.26 [0.26; 0.26] |
| 60-64 years | 582 | 1447 | 109 | 0.21 [0.21; 0.21] |
| 65+ years | 323 | 953 | 55 | 0.17 [0.17; 0.17] |
| **Neighborhood income** |  |  |  |  |
| Tertile 1 (high) | 4670 | 11,875 | 1956 | 0.52 [0.51; 0.53] |
| Tertile 2 | 5201 | 11,988 | 2286 | 0.61 [0.60; 0.63] |
| Tertile 3 (low) | 4589 | 11,612 | 2184 | 0.60 [0.58; 0.61] |
| **Neighborhood welfare** |  |  |  |  |
| Tertile 1 (low) | 4699 | 11,930 | 1965 | 0.52 [0.51; 0.53] |
| Tertile 2 | 5031 | 11,762 | 2266 | 0.64 [0.62; 0.64] |
| Tertile 3 (high) | 4294 | 11,784 | 2195 | 0.59 [0.58; 0.60] |
| **Neighborhood crime** |  |  |  |  |
| Tertile 1 (low) | 5318 | 11,920 | 2008 | 0.52 [0.51; 0.53] |
| Tertile 2 | 5980 | 11,903 | 2146 | 0.56 [0.55; 0.57] |
| Tertile 3 (high) | 5247 | 11,653 | 2272 | 0.66 [0.64; 0.67] |

Note: Violent reoffending rates were estimated using negative binomial regression models to adjust for overdispersion.

**ST5. Socio-demographic data on released prisoners with diagnoses of alcohol use disorder.**

|  | **Number of individuals** | **Number of person-years at risk** | **Number of violent crimes** | **Violent reoffending**  **rate per person-year**  **[95% CI]** |
| --- | --- | --- | --- | --- |
| **Total** | 15,103 | 66,540 | 14,565 | 0.67 [0.66; 0.67] |
| **Sex** |  |  |  |  |
| Male | 13,679 | 59,907 | 14,013 | 0.72 [0.72; 0.72] |
| Female | 1424 | 6635 | 552 | 0.19 [0.19; 0.19] |
| **Age groups** |  |  |  |  |
| 15-19 years | 228 | 194 | 141 | 2.09 [2.09; 2.09] |
| 20-24 years | 2062 | 3985 | 1869 | 1.63 [1.63; 1.63] |
| 25-29 years | 2827 | 6556 | 2003 | 1.27 [1.27; 1.27] |
| 30-34 years | 2776 | 6601 | 1934 | 0.99 [0.99; 0.99] |
| 35-39 years | 3008 | 6876 | 2002 | 0.77 [0.77; 0.77] |
| 40-44 years | 3606 | 8708 | 1998 | 0.60 [0.60; 0.60] |
| 45-49 years | 4098 | 10,172 | 2061 | 0.47 [0.47; 0.47] |
| 50-54 years | 3642 | 9219 | 1334 | 0.37 [0.37; 0.37] |
| 55-59 years | 2690 | 6785 | 753 | 0.29 [0.29; 0.29] |
| 60-64 years | 1734 | 4352 | 329 | 0.22 [0.22; 0.22] |
| 65+ years | 1013 | 3094 | 142 | 0.17 [0.17; 0.17] |
| **Neighborhood income** |  |  |  |  |
| Tertile 1 (high) | 8784 | 22,201 | 4549 | 0.61 [0.60; 0.62] |
| Tertile 2 | 9731 | 22,565 | 5126 | 0.70 [0.69; 0.71] |
| Tertile 3 (low) | 8674 | 21,777 | 4891 | 0.70 [0.69; 0.71] |
| **Neighborhood welfare** |  |  |  |  |
| Tertile 1 (low) | 8839 | 22,425 | 4200 | 0.54 [0.53; 0.55] |
| Tertile 2 | 9484 | 22,048 | 5200 | 0.76 [0.75; 0.77] |
| Tertile 3 (high) | 8247 | 22,070 | 5166 | 0.71 [0.70; 0.72] |
| **Neighborhood crime** |  |  |  |  |
| Tertile 1 (low) | 9962 | 22,313 | 4590 | 0.62 [0.61; 0.63] |
| Tertile 2 | 11,278 | 22,347 | 4826 | 0.68 [0.67; 0.69] |
| Tertile 3 (high) | 9936 | 21,883 | 5150 | 0.70 [0.69; 0.71] |

Note: Violent reoffending rates were estimated using negative binomial regression models to adjust for overdispersion.

**ST6. Socio-demographic data on released prisoners with diagnoses of drug use disorder.**

|  | **Number of individuals** | **Number of person-years at risk** | **Number of violent crimes** | **Violent reoffending**  **rate per person-year**  **[95% CI]** |
| --- | --- | --- | --- | --- |
| **Total** | 16,164 | 72,414 | 17,271 | 0.70 [0.69; 0.70] |
| **Sex** |  |  |  |  |
| Male | 14,340 | 63,496 | 16,661 | 0.77 [0.77; 0.77] |
| Female | 1824 | 8918 | 610 | 0.13 [0.13; 0.13] |
| **Age groups** |  |  |  |  |
| 15-19 years | 409 | 339 | 248 | 1.65 [1.65; 1.65] |
| 20-24 years | 3536 | 6648 | 3236 | 1.31 [1.31; 1.31] |
| 25-29 years | 4878 | 11584 | 3449 | 1.04 [1.04; 1.04] |
| 30-34 years | 4503 | 10818 | 2679 | 0.82 [0.82; 0.82] |
| 35-39 years | 4117 | 9462 | 2299 | 0.65 [0.65; 0.65] |
| 40-44 years | 4159 | 10,239 | 1998 | 0.52 [0.52; 0.52] |
| 45-49 years | 3988 | 10,141 | 1741 | 0.41 [0.41; 0.41] |
| 50-54 years | 2839 | 7152 | 1058 | 0.33 [0.33; 0.33] |
| 55-59 years | 1561 | 3826 | 386 | 0.26 [0.26; 0.26] |
| 60-64 years | 658 | 1568 | 142 | 0.21 [0.21; 0.21] |
| 65+ years | 233 | 639 | 36 | 0.16 [0.16; 0.16] |
| **Neighborhood income** |  |  |  |  |
| Tertile 1 (high) | 9664 | 23,952 | 5513 | 0.65 [0.65; 0.65] |
| Tertile 2 | 10,613 | 24,630 | 6206 | 0.78 [0.77; 0.79] |
| Tertile 3 (low) | 9695 | 23,832 | 5553 | 0.67 [0.66; 0.68] |
| **Neighborhood welfare** |  |  |  |  |
| Tertile 1 (low) | 9776 | 24,376 | 5192 | 0.62 [0.62; 0.63] |
| Tertile 2 | 10,462 | 24,052 | 6137 | 0.74 [0.73; 0.75] |
| Tertile 3 (high) | 9039 | 23,986 | 5943 | 0.73 [0.73; 0.74] |
| **Neighborhood crime** |  |  |  |  |
| Tertile 1 (low) | 10,635 | 24,432 | 5315 | 0.60 [0.60; 0.61] |
| Tertile 2 | 12,222 | 24,257 | 5910 | 0.75 [0.74; 0.75] |
| Tertile 3 (high) | 10,844 | 23,726 | 6047 | 0.75 [0.74; 0.76] |

Note: Violent reoffending rates were estimated using negative binomial regression models to adjust for overdispersion.

**ST7. Socio-demographic data on released prisoners that have been prescribed antipsychotics.**

|  | **Number of individuals** | **Number of person-years at risk** | **Number of violent crimes** | **Violent reoffending**  **rate per person-year**  **[95% CI]** |
| --- | --- | --- | --- | --- |
| **Total** | 7366 | 31,458 | 8295 | 0.82 [0.81; 0.83] |
| **Sex** |  |  |  |  |
| Male | 6543 | 27,889 | 7987 | 0.90 [0.90; 0.90] |
| Female | 823 | 3568 | 308 | 0.18 [0.18; 0.18] |
| **Age groups** |  |  |  |  |
| 15-19 years | 170 | 140 | 112 | 1.77 [1.77; 1.77] |
| 20-24 years | 1622 | 3063 | 1666 | 1.43 [1.43; 1.43] |
| 25-29 years | 2233 | 5251 | 1677 | 1.16 [1.16; 1.16] |
| 30-34 years | 2094 | 4973 | 1430 | 0.93 [0.93; 0.93] |
| 35-39 years | 1850 | 4348 | 1121 | 0.75 [0.75; 0.75] |
| 40-44 years | 1753 | 4276 | 830 | 0.61 [0.61; 0.61] |
| 45-49 years | 1586 | 3923 | 737 | 0.49 [0.49; 0.49] |
| 50-54 years | 1062 | 2715 | 426 | 0.40 [0.40; 0.40] |
| 55-59 years | 616 | 1518 | 191 | 0.32 [0.32; 0.32] |
| 60-64 years | 301 | 722 | 89 | 0.26 [0.26; 0.26] |
| 65+ years | 162 | 528 | 16 | 0.21 [0.21; 0.21] |
| **Neighborhood income** |  |  |  |  |
| Tertile 1 (high) | 4322 | 10,498 | 2537 | 0.72 [0.71; 0.74] |
| Tertile 2 | 4748 | 10,682 | 2985 | 0.92 [0.91; 0.94] |
| Tertile 3 (low) | 4266 | 10,278 | 2773 | 0.83 [0.81; 0.84] |
| **Neighborhood welfare** |  |  |  |  |
| Tertile 1 (low) | 4346 | 10,609 | 2548 | 0.71 [0.70; 0.73] |
| Tertile 2 | 4645 | 10,398 | 3029 | 0.95 [0.94; 0.97] |
| Tertile 3 (high) | 3955 | 10,451 | 2718 | 0.81 [0.80; 0.83] |
| **Neighborhood crime** |  |  |  |  |
| Tertile 1 (low) | 4793 | 10,607 | 2661 | 0.71 [0.70; 0.73] |
| Tertile 2 | 5434 | 10,528 | 2900 | 0.95 [0.94; 0.97] |
| Tertile 3 (high) | 4709 | 10,323 | 2734 | 0.81 [0.80; 0.83] |

Note: Violent reoffending rates were estimated using negative binomial regression models to adjust for overdispersion.

**ST8. Socio-demographic data on released prisoners that have been prescribed mood stabilizers**

|  | **Number of individuals** | **Number of person-years at risk** | **Number of violent crimes** | **Violent reoffending**  **rate per person-year**  **[95% CI]** |
| --- | --- | --- | --- | --- |
| **Total** | 3796 | 16,184 | 4057 | 0.72 [0.71; 0.73] |
| **Sex** |  |  |  |  |
| Male | 3333 | 14,200 | 3860 | 0.79 [0.79; 0.79] |
| Female | 463 | 1985 | 197 | 0.21 [0.21; 0.21] |
| **Age groups** |  |  |  |  |
| 15-19 years | 69 | 58 | 46 | 1.35 [1.35; 1.35] |
| 20-24 years | 731 | 1433 | 740 | 1.15 [1.15; 1.15] |
| 25-29 years | 1032 | 2424 | 699 | 0.99 [0.99; 0.99] |
| 30-34 years | 951 | 2229 | 591 | 0.84 [0.84; 0.84] |
| 35-39 years | 889 | 2053 | 520 | 0.72 [0.72; 0.72] |
| 40-44 years | 902 | 2139 | 469 | 0.62 [0.62; 0.62] |
| 45-49 years | 883 | 2176 | 433 | 0.53 [0.53; 0.53] |
| 50-54 years | 673 | 1708 | 297 | 0.45 [0.45; 0.45] |
| 55-59 years | 432 | 1039 | 164 | 0.38 [0.38; 0.38] |
| 60-64 years | 247 | 577 | 69 | 0.33 [0.33; 0.33] |
| 65+ years | 114 | 347 | 29 | 0.28 [0.28; 0.28] |
| **Neighborhood income** |  |  |  |  |
| Tertile 1 (high) | 2235 | 5401 | 1218 | 0.63 [0.61; 0.65] |
| Tertile 2 | 2505 | 5480 | 1460 | 0.77 [0.75; 0.79] |
| Tertile 3 (low) | 2219 | 5303 | 1379 | 0.77 [0.75; 0.79] |
| **Neighborhood welfare** |  |  |  |  |
| Tertile 1 (low) | 2268 | 5468 | 1235 | 0.66 [0.64; 0.68] |
| Tertile 2 | 2420 | 5361 | 1466 | 0.80 [0.78; 0.82] |
| Tertile 3 (high) | 2086 | 5355 | 1356 | 0.71 [0.69; 0.73] |
| **Neighborhood crime** |  |  |  |  |
| Tertile 1 (low) | 2509 | 5475 | 1210 | 0.62 [0.60; 0.64] |
| Tertile 2 | 2813 | 5391 | 1375 | 0.72 [0.70; 0.74] |
| Tertile 3 (high) | 2470 | 5317 | 1472 | 0.83 [0.81; 0.85] |

Note: Violent reoffending rates were estimated using negative binomial regression models to adjust for overdispersion.

**ST9. Socio-demographic data on released prisoners that have been prescribed anxiolytics**

|  | **Number of individuals** | **Number of person-years at risk** | **Number of violent crimes** | **Violent reoffending**  **rate per person-year**  **[95% CI]** |
| --- | --- | --- | --- | --- |
| **Total** | 17,137 | 76,513 | 13,398 | 0.55 [0.54; 0.55] |
| **Sex** |  |  |  |  |
| Male | 15,053 | 66,753 | 12,849 | 0.61 [0.61; 0.61] |
| Female | 2084 | 9760 | 549 | 0.11 [0.11; 0.11] |
| **Age groups** |  |  |  |  |
| 15-19 years | 291 | 272 | 153 | 1.52 [1.52; 1.52] |
| 20-24 years | 2979 | 5856 | 2214 | 1.18 [1.18; 1.18] |
| 25-29 years | 4309 | 10,577 | 2399 | 0.91 [0.91; 0.91] |
| 30-34 years | 4158 | 9977 | 2086 | 0.71 [0.71; 0.71] |
| 35-39 years | 3950 | 9501 | 1859 | 0.55 [0.55; 0.55] |
| 40-44 years | 4174 | 10,454 | 1673 | 0.42 [0.42; 0.42] |
| 45-49 years | 4183 | 10,721 | 1451 | 0.33 [0.33; 0.33] |
| 50-54 years | 3254 | 8323 | 914 | 0.25 [0.25; 0.25] |
| 55-59 years | 2104 | 5318 | 394 | 0.20 [0.20; 0.20] |
| 60-64 years | 1261 | 3105 | 181 | 0.15 [0.15; 0.15] |
| 65+ years | 739 | 2409 | 74 | 0.12 [0.12; 0.12] |
| **Neighborhood income** |  |  |  |  |
| Tertile 1 (high) | 9882 | 25,472 | 4181 | 0.50 [0.49; 0.51] |
| Tertile 2 | 10,957 | 25,828 | 4672 | 0.58 [0.57; 0.59] |
| Tertile 3 (low) | 9786 | 25,213 | 4545 | 0.56 [0.55; 0.57] |
| **Neighborhood welfare** |  |  |  |  |
| Tertile 1 (low) | 9989 | 25,740 | 4051 | 0.48 [0.47; 0.49] |
| Tertile 2 | 10,680 | 25,486 | 4756 | 0.57 [0.56; 0.58] |
| Tertile 3 (high) | 9097 | 25,287 | 4591 | 0.58 [0.58; 0.59] |
| **Neighborhood crime** |  |  |  |  |
| Tertile 1 (low) | 11,189 | 25,722 | 4252 | 0.50 [0.49; 0.50] |
| Tertile 2 | 12,739 | 25,550 | 4537 | 0.56 [0.55; 0.56] |
| Tertile 3 (high) | 11,171 | 25,241 | 4609 | 0.59 [0.58; 0.59] |

Note: Violent reoffending rates were estimated using negative binomial regression models to adjust for overdispersion.

**ST10. Socio-demographic data on released prisoners that have been prescribed antidepressants**

|  | **Number of individuals** | **Number of person-years at risk** | **Number of violent crimes** | **Violent reoffending**  **rate per person-year**  **[95% CI]** |
| --- | --- | --- | --- | --- |
| **Total** | 17,836 | 79,031 | 13,998 | 0.55 [0.55; 0.56] |
| **Sex** |  |  |  |  |
| Male | 15,764 | 69,421 | 13,490 | 0.61 [0.61; 0.61] |
| Female | 2072 | 9610 | 9610 | 0.10 [0.10; 0.10] |
| **Age groups** |  |  |  |  |
| 15-19 years | 358 | 315 | 197 | 1.41 [1.41; 1.41] |
| 20-24 years | 3333 | 6541 | 2580 | 1.10 [1.10; 1.10] |
| 25-29 years | 4800 | 11741 | 2611 | 0.86 [0.86; 0.86] |
| 30-34 years | 4571 | 11126 | 2273 | 0.68 [0.68; 0.68] |
| 35-39 years | 4155 | 10084 | 1890 | 0.53 [0.53; 0.53] |
| 40-44 years | 4324 | 10856 | 1601 | 0.42 [0.42; 0.42] |
| 45-49 years | 4197 | 10754 | 1455 | 0.33 [0.33; 0.33] |
| 50-54 years | 3090 | 7870 | 779 | 0.26 [0.26; 0.26] |
| 55-59 years | 1930 | 4935 | 363 | 0.20 [0.20; 0.20] |
| 60-64 years | 1112 | 2776 | 166 | 0.16 [0.16; 0.16] |
| 65+ years | 644 | 2035 | 83 | 0.12 [0.12; 0.12] |
| **Neighborhood income** |  |  |  |  |
| Tertile 1 (high) | 11,629 | 26,581 | 4524 | 0.48 [0.48; 0.49] |
| Tertile 2 | 13,196 | 26,495 | 4631 | 0.59 [0.58; 0.60] |
| Tertile 3 (low) | 11,609 | 25,956 | 4843 | 0.59 [0.58; 0.60] |
| **Neighborhood welfare** |  |  |  |  |
| Tertile 1 (low) | 10,365 | 26,611 | 4249 | 0.50 [0.49; 0.50] |
| Tertile 2 | 11,046 | 26,240 | 5061 | 0.61 [0.60; 0.61] |
| Tertile 3 (high) | 9507 | 26,180 | 4688 | 0.56 [0.55; 0.57] |
| **Neighborhood crime** |  |  |  |  |
| Tertile 1 (low) | 10,333 | 26,415 | 4228 | 0.52 [0.51; 0.53] |
| Tertile 2 | 11,491 | 26,680 | 4870 | 0.55 [0.54; 0.55] |
| Tertile 3 (high) | 10,077 | 25,937 | 4900 | 0.60 [0.59; 0.60] |

Note: Violent reoffending rates were estimated using negative binomial regression models to adjust for overdispersion.

**ST11. Socio-demographic data on released prisoners that have been prescribed drugs used in alcohol dependence**

|  | **Number of individuals** | **Number of person-years at risk** | **Number of violent crimes** | **Violent reoffending**  **rate per person-year**  **[95% CI]** |
| --- | --- | --- | --- | --- |
| **Total** | 6416 | 27,748 | 6402 | 0.71 [0.70; 0.72] |
| **Sex** |  |  |  |  |
| Male | 5769 | 24,938 | 6159 | 0.76 [0.76; 0.76] |
| Female | 647 | 2809 | 233 | 0.20 [0.20; 0.20] |
| **Age groups** |  |  |  |  |
| 15-19 years | 58 | 46 | 50 | 2.64 [2.64; 2.64] |
| 20-24 years | 762 | 1462 | 716 | 1.96 [1.96; 1.96] |
| 25-29 years | 1169 | 2676 | 892 | 1.46 [1.46; 1.46] |
| 30-34 years | 1270 | 2906 | 992 | 1.09 [1.09; 1.09] |
| 35-39 years | 1385 | 3264 | 990 | 0.81 [0.81; 0.81] |
| 40-44 years | 1707 | 4137 | 1009 | 0.60 [0.60; 0.60] |
| 45-49 years | 1810 | 4457 | 899 | 0.45 [0.45; 0.45] |
| 50-54 years | 1446 | 3616 | 481 | 0.33 [0.33; 0.33] |
| 55-59 years | 1025 | 2598 | 248 | 0.25 [0.25; 0.25] |
| 60-64 years | 615 | 1564 | 95 | 0.19 [0.19; 0.19] |
| 65+ years | 359 | 1021 | 30 | 0.14 [0.14; 0.14] |
| **Neighborhood income** |  |  |  |  |
| Tertile 1 (high) | 3703 | 9305 | 1982 | 0.65 [0.64; 0.66] |
| Tertile 2 | 4167 | 9353 | 2227 | 0.71 [0.70; 0.73] |
| Tertile 3 (low) | 3684 | 9090 | 2193 | 0.76 [0.75; 0.78] |
| **Neighborhood welfare** |  |  |  |  |
| Tertile 1 (low) | 3830 | 9341 | 1879 | 0.59 [0.57; 0.60] |
| Tertile 2 | 4053 | 9198 | 2215 | 0.77 [0.75; 0.78] |
| Tertile 3 (high) | 3500 | 9209 | 2308 | 0.78 [0.76; 0.79] |
| **Neighborhood crime** |  |  |  |  |
| Tertile 1 (low) | 4397 | 9306 | 2075 | 0.68 [0.67; 0.70] |
| Tertile 2 | 4781 | 9296 | 1944 | 0.62 [0.61; 0.64] |
| Tertile 3 (high) | 4259 | 9146 | 2383 | 0.82 [0.80; 0.83] |

Note: Violent reoffending rates were estimated using negative binomial regression models to adjust for overdispersion.

**ST12. Socio-demographic data on released prisoners that have been prescribed drugs used in opioid dependence**

|  | **Number of individuals** | **Number of person-years at risk** | **Number of violent crimes** | **Violent reoffending**  **rate per person-year**  **[95% CI]** |
| --- | --- | --- | --- | --- |
| **Total** | 2172 | 11,107 | 1862 | 0.47 [0.46; 0.48] |
| **Sex** |  |  |  |  |
| Male | 1868 | 9593 | 1795 | 0.53 [0.53; 0.53] |
| Female | 304 | 1514 | 67 | 0.08 [0.08; 0.08] |
| **Age groups** |  |  |  |  |
| 15-19 years | 17 | 15 | 13 | 1.42 [1.42; 1.42] |
| 20-24 years | 363 | 689 | 234 | 1.02 [1.02; 1.02] |
| 25-29 years | 812 | 2081 | 469 | 0.73 [0.73; 0.73] |
| 30-34 years | 937 | 2438 | 509 | 0.52 [0.52; 0.52] |
| 35-39 years | 767 | 1864 | 330 | 0.37 [0.37; 0.37] |
| 40-44 years | 610 | 1629 | 148 | 0.27 [0.27; 0.27] |
| 45-49 years | 490 | 1306 | 98 | 0.19 [0.19; 0.19] |
| 50-54 years | 289 | 751 | 43 | 0.14 [0.14; 0.14] |
| 55-59 years | 101 | 240 | 14 | 0.10 [0.10; 0.10] |
| 60-64 years | 30 | 66 | 4 | 0.07 [0.07; 0.07] |
| 65+ years | 10 | 29 | 0 | 0.05 [0.05; 0.05] |
| **Neighborhood income** |  |  |  |  |
| Tertile 1 (high) | 1288 | 3699 | 605 | 0.42 [0.40; 0.44] |
| Tertile 2 | 1443 | 3769 | 645 | 0.56 [0.54; 0.58] |
| Tertile 3 (low) | 1329 | 3639 | 612 | 0.45 [0.44; 0.47] |
| **Neighborhood welfare** |  |  |  |  |
| Tertile 1 (low) | 1349 | 3779 | 589 | 0.40 [0.39; 0.42] |
| Tertile 2 | 1409 | 3695 | 647 | 0.59 [0.57; 0.61] |
| Tertile 3 (high) | 1221 | 3634 | 626 | 0.44 [0.43; 0.46] |
| **Neighborhood crime** |  |  |  |  |
| Tertile 1 (low) | 1514 | 3760 | 583 | 0.41 [0.39; 0.43] |
| Tertile 2 | 1712 | 3721 | 632 | 0.52 [0.50; 0.54] |
| Tertile 3 (high) | 1434 | 3627 | 647 | 0.50 [0.48; 0.52] |

Note: Violent reoffending rates were estimated using negative binomial regression models to adjust for overdispersion.

**ST13. Crude intra-class correlations (ICC) for alternative geographical representations in released prisoners stratified across all released prisoners, prisoners with psychiatric and substance use disorder, and prisoners that have been prescribed medications for psychiatric and substance use disorders**

|  | **Neighborhoods** | **Parishes** | **Municipalities** | **Counties** |
| --- | --- | --- | --- | --- |
|  | **ICC [95% CI]** | **ICC [95% CI]** | **ICC [95% CI]** | **ICC [95% CI]** |
| **All prisoners** | 4.3% [3.7%; 4.9%] | 2.0% [1.6%; 2.5%] | 1.1% [0.8%; 1.5%] | 0.4% [0.2%; 0.9%] |
|  |  |  |  |  |
| **Psychiatric disorders** |  |  |  |  |
| Any psychotic disorders | 13.5% [10.9%; 16.6%] | 4.7% [3.1%; 7.1%] | 2.3% [1.2%; 4.3%] | 0.4% [0.1%; 1.2%] |
| Personality disorder | 11.0% [9.1%; 13.3%] | 3.8% [2.6%; 5.4%] | 2.0% [1.2%; 3.4%] | 0.7% [0.3%; 1.6%] |
| Anxiety | 7.3% [5.9%; 9.1%] | 3.2% [2.3%; 4.4%] | 1.9% [1.2%; 2.8%] | 0.9% [0.4%; 1.9%] |
| Depression | 8.7% [6.9%; 11.0%] | 3.2% [2.1%; 4.6%] | 2.1% [1.3%; 3.4%] | 0.7% [0.3%; 1.5%] |
| Alcohol use disorder | 7.6% [6.5%; 8.9%] | 2.7% [2.0%; 3.6%] | 1.4% [0.9%; 2.1%] | 0.5% [0.2%; 1.1%] |
| Drug use disorder | 5.5% [4.6%; 6.5%] | 2.4% [1.9%; 3.2%] | 1.7% [1.2%; 2.5%] | 0.6% [0.3%; 1.2%] |
|  |  |  |  |  |
| **Individuals prescribed psychiatric medications** |  |  |  |  |
| Antipsychotics | 6.9% [5.4%; 8.7%] | 2.4% [1.6%; 3.6%] | 1.4% [0.8%; 2.4%] | 0.5% [0.2%; 1.2%] |
| Mood stabilizers | 10.3% [8.0%; 13.2%] | 4.8% [3.3%; 6.8%] | 3.1% [1.9%; 4.9%] | 0.7% [0.2%; 1.8%] |
| Anxiolytics | 6.7% [5.7%; 8.0%] | 2.7% [2.1%; 3.6%] | 1.8% [1.3%; 2.6%] | 0.8% [0.4%; 1.6%] |
| Antidepressants | 6.0% [5.0%; 7.2%] | 2.4% [1.8%; 3.2%] | 1.6% [1.1%; 2.3%] | 0.8% [0.4%; 1.6%] |
| Drugs used in alcohol dependence | 6.8% [5.1%; 9.0%] | 1.9% [1.1%; 3.2%] | 1.1% [0.6%; 2.0%] | 0.2% [0.1%; 0.9%] |
| Drugs used in opioid dependence | 10.3% [7.0%; 15.0%] | 5.2% [3.0%; 8.8%] | 3.9% [1.9%; 7.8%] | 1.0% [0.3%; 3.6%] |

**ST14. Sex, ethnicity, and population size-specific incidence rate ratios (IRRs) for the associations between the neighborhood-level variables and violent reoffending in all released prisoners.**

|  | **Males only** | | **No immigrant background** | | **At least 1500 neighborhood residents** | |
| --- | --- | --- | --- | --- | --- | --- |
|  | **Model I** | **Model II** | **Model I** | **Model II** | **Model I** | **Model II** |
|  | **IRR**  **[95% CI]** | **IRR**  **[95% CI]** | **IRR**  **[95% CI]** | **IRR**  **[95% CI]** | **IRR**  **[95% CI]** | **IRR**  **[95% CI]** |
| Neighborhood income | 1.07  [1.03; 1.11] | 0.97  [0.92; 1.02] | 1.06  [1.00; 1.12] | 1.00  [0.93; 1.07] | 1.05  [1.01; 1.10] | 0.97  [0.91; 1.04] |
| Neighborhood welfare | 1.06  [1.02; 1.10] | 0.98  [0.93; 1.05] | 1.13  [1.06; 1.20] | 0.94  [0.86; 1.03] | 1.04  [1.00; 1.09] | 0.99  [0.92; 1.08] |
| Neighborhood crime | 1.09  [1.05; 1.13] | 1.02  [0.98; 1.07] | 1.13  [1.07; 1.19] | 1.00  [0.94; 1.06] | 1.06  [1.00; 1.12] | 1.02  [0.94; 1.10] |

*Notes: Neighborhood income was reverse-coded (e.g., the estimates refer to the effects on violent reoffending risk following a standardized unit reduction of the neighborhood income). Model I: Crude between-estimate; Model II: Within-individual estimate, adjusted for age.*

**ST15. Within-individual correlations (ICC) for neighborhood factors, stratified across all released prisoners, prisoners with psychiatric and substance use disorder, and prisoners that have been prescribed medications for psychiatric and substance use disorders**

|  | **Neighborhood income** | **Neighborhood welfare** | **Neighborhood crime** |
| --- | --- | --- | --- |
|  | **ICC**  **[95% CI]** | **ICC**  **[95% CI]** | **ICC**  **[95% CI]** |
| **All prisoners** | 0.59 [0.59; 0.60] | 0.68 [0.67; 0.68] | 0.37 [0.37; 0.37] |
|  |  |  |  |
| **Psychiatric disorders** |  |  |  |
| Any psychotic disorders | 0.54 [0.52; 0.55] | 0.62 [0.61; 0.64] | 0.35 [0.34; 0.37] |
| Personality disorder | 0.52 [0.51; 0.54] | 0.61 [0.60; 0.62] | 0.33 [0.32; 0.35] |
| Anxiety | 0.54 [0.53; 0.55] | 0.63 [0.62; 0.64] | 0.31 [0.30; 0.32] |
| Depression | 0.55[0.54; 0.56] | 0.61 [0.60; 0.62] | 0.28 [0.27; 0.30] |
| Alcohol use disorder | 0.54 [0.54; 0.55] | 0.61 [0.61; 0.62] | 0.33 [0.32; 0.34] |
| Drug use disorder | 0.53 [0.52; 0.53] | 0.60 [0.60; 0.61] | 0.33 [0.32; 0.33] |
|  |  |  |  |
| **Individuals prescribed psychiatric medications** |  |  |  |
| Antipsychotics | 0.55 [0.54; 0.56] | 0.63 [0.62; 0.64] | 0.32 [0.31; 0.33] |
| Mood stabilizers | 0.51 [0.50; 0.53] | 0.59 [0.58; 0.61] | 0.28 [0.27; 0.30] |
| Anxiolytics | 0.57 [0.57; 0.58] | 0.65 [0.64; 0.66] | 0.35 [0.34; 0.36] |
| Antidepressants | 0.56 [0.55; 0.56] | 0.65 [0.64; 0.65] | 0.33 [0.32; 0.33] |
| Drugs used in alcohol dependence | 0.51 [0.50; 0.52] | 0.58 [0.57; 0.59] | 0.27 [0.26; 0.28] |
| Drugs used in opioid dependence | 0.56 [0.55; 0.56] | 0.58 [0.56; 0.59] | 0.33 [0.31; 0.35] |

**ST16. Incidence rate ratios (IRRs) for the associations between neighborhood factors (averaged across 2002-2012) and violent reoffending stratified across all released prisoners, prisoners with psychiatric and substance use disorder, and prisoners that have been prescribed medications for psychiatric and substance use disorders**

|  | **Mean neighborhood income** | | **Mean neighborhood welfare** | | **Mean neighborhood crime** | |
| --- | --- | --- | --- | --- | --- | --- |
|  | **Model I** | **Model II** | **Model I** | **Model II** | **Model I** | **Model II** |
|  | **IRR**  **[95% CI]** | **IRR**  **[95% CI]** | **IRR**  **[95% CI]** | **IRR**  **[95% CI]** | **IRR**  **[95% CI]** | **IRR**  **[95% CI]** |
| **All prisoners** | 1.01 [0.97; 1.05] | 0.95 [0.89; 1.01] | 1.06 [1.02; 1.10] | 0.98 [0.91; 1.04] | 1.09 [1.04; 1.13] | 1.02 [0.96; 1.09] |
| **Psychiatric disorders** |  |  |  |  |  |  |
| Any psychotic disorders | 0.97 [0.86; 1.09] | 0.91 [0.78; 1.06] | 1.11 [0.98; 1.25] | 0.93 [0.79; 1.09] | 1.11 [0.99; 1.26] | 1.01 [0.86; 1.19] |
| Personality disorder | 0.97 [0.89; 1.06] | 1.01 [0.88; 1.15] | 1.10 [1.01; 1.20] | 0.85 [0.75; 0.97] | 1.11 [1.01; 1.22] | 0.94 [0.81; 1.08] |
| Anxiety | 1.00 [0.93; 1.07] | 0.90 [0.80; 1.01] | 1.05 [0.98; 1.12] | 0.97 [0.86; 1.09] | 1.07 [1.00; 1.16] | 0.98 [0.87; 1.10] |
| Depression | 0.95 [0.88; 1.04] | 1.00 [0.88; 1.14] | 1.08 [0.99; 1.18] | 0.89 [0.79; 1.01] | 1.10 [1.01; 1.20] | 0.91[0.79; 1.05] |
| Alcohol use disorder | 1.02 [0.96; 1.08] | 0.94 [0.85; 1.03] | 1.07 [1.00; 1.15] | 0.95 [0.86; 1.05] | 1.05 [0.98; 1.12] | 1.02 [0.93; 1.12] |
| Drug use disorder | 0.98 [0.93; 1.03] | 0.99 [0.91; 1.07] | 1.04 [0.99; 1.10] | 0.96 [0.89; 1.04] | 1.05 [1.00; 1.11] | 1.02 [0.94; 1.10] |
| **Individuals prescribed psychiatric medications** |  |  |  |  |  |  |
| Antipsychotics | 0.98 [0.91; 1.05] | 0.93 [0.83; 1.04] | 1.02 [0.95; 1.09] | 1.01 [0.89; 1.13] | 1.05 [0.98; 1.14] | 0.99 [0.88; 1.13] |
| Mood stabilizers | 0.98 [0.87; 1.10] | 1.07 [0.91; 1.26] | 1.01 [0.91; 1.13] | 0.91 [0.77; 1.08] | 1.03 [0.92; 1.17] | 0.90 [0.76; 1.06] |
| Anxiolytics | 1.00 [0.94; 1.06] | 0.94 [0.85; 1.02] | 1.04 [0.98; 1.10] | 0.97 [0.88; 1.07] | 1.06 [1.00; 1.12] | 1.02 [0.92; 1.11] |
| Antidepressants | 1.02 [0.96; 1.08] | 0.92 [0.84; 1.01] | 1.01 [0.95; 1.07] | 0.96 [0.87; 1.06] | 1.05 [0.99; 1.11] | 1.00 [0.91; 1.10] |
| Drugs used in alcohol dependence | 1.00 [0.91; 1.10] | 0.93 [0.81; 1.06] | 1.14 [1.03; 1.27] | 0.93 [0.80; 1.09] | 1.11 [1.00; 1.24] | 1.04 [0.91; 1.20] |
| Drugs used in opioid dependence | 0.90 [0.81; 1.00] | 0.99 [0.84; 1.15] | 1.07 [0.96; 1.19] | 0.99 [0.84; 1.16] | 1.10 [0.97; 1.24] | 0.97[0.81; 1.16] |

*Notes: Mean neighborhood income was reverse-coded (e.g., the estimates refer to the effects on violent reoffending risk following a standardized unit reduction of the mean neighborhood income). Model I: Crude between-estimate; Model II: Within-individual estimate, adjusted for age.*
